# Occurrence of selected endocrine disrupting compounds in the eastern cape province of South Africa

**DOI:** 10.1007/s11356-020-08082-y

**Published:** 2020-03-09

**Authors:** Adebayo I. Farounbi, Nosiphiwe P. Ngqwala

**Affiliations:** Environmental Health and Biotechnology Research Group, Division of Pharmaceutical Chemistry, Faculty of Pharmacy, Rhodes University, P.O. Box 94, Grahamstown, 6140 South Africa

**Keywords:** Endocrine disruptor, Liquid chromatography, Solid phase extraction, Freshwater, Wastewater, Health

## Abstract

Endocrine-disrupting compounds are attracting attention worldwide because of their effects on living things in the environment. Ten endocrine disrupting compounds: 4-nonylphenol, 2,4-dichlorophenol, estrone, 17β-estradiol, bisphenol A, 4-tert-octylphenol, triclosan, atrazine, imidazole and 1,2,4-triazole were investigated in four rivers and wastewater treatment plants in this study. Rivers were sampled at upstream, midstream and downstream reaches, while the influent and effluent samples of wastewater were collected from treatment plants near the receiving rivers. Sample waters were freeze-dried followed by extraction of the organic content and purification by solid-phase extraction. Concentrations of the compounds in the samples were determined with ultra-high performance liquid chromatography-tandem mass spectrometry. The instrument was operated in the positive electrospray ionization (ESI) mode. The results showed that these compounds are present in the samples with nonylphenol > dichlorophenol > bisphenol A > triclosan > octylphenol > imidazole > atrazine > triazole > estrone > estradiol. Nonylphenol has its highest concentration of 6.72 μg/L in King Williams Town wastewater influent and 2.55 μg/L in midstream Bloukrans River. Dichlorophenol has its highest concentration in Alice wastewater influent with 2.20 μg/L, while it was 0.737 μg/L in midstream Bloukrans River. Uitenhage wastewater effluent has bisphenol A concentration of 1.684 μg/L while it was 0.477 μg/L in the downstream samples of the Bloukrans River. Generally, the upstream samples of the rivers had lesser concentrations of the compounds. The wastewater treatment plants were not able to achieve total removal of the compounds in the wastewater while runoffs and wastes dump from the cities contributed to the concentrations of the compounds in the rivers.

## Introduction

The endocrine system, in the body of vertebrates, is made up of organs that regulate essential functions such as reproduction, metabolism, water balance, feeding and growth. The knowledge of the endocrine system dates back to pre-historic times when animal testes were removed to make them sterile and fatter and male humans serving in palaces were castrated to eunuchs (Darbre 2019). The ancient Chinese since 1600 BC used seaweed and heated sponges to treat goitre, a problem caused by iodine deficiency in the thyroid gland (Kleine and Rossmanith 2016). Endocrine glands produce hormones in quantities and qualities adequate to communicate, synchronize and ensure the normal functioning of the whole body system. An endocrine-disrupting compound (EDC) is any chemical, natural or synthetic that can mimic, obstruct the binding site of a hormone or prevent the production and effects of such hormone (Lee et al. 2013). Such compound can be of natural or artificial origin. They have the ability to cause temporary or permanent health problems in normal organisms or their progenies at nanogram concentrations (Stolz et al. 2018; Pal et al. 2014). There are about one thousand compounds identified with endocrine disruption abilities and their numbers are growing (Gore et al. 2014). Out of these compounds, very few have been investigated and documented (WHO 2012). Since they are emerging contaminants, the activities of some potential endocrine disruptors are yet to be determined. The effects of EDCs may result in either under-function or over-function of the endocrine system. Any of these may result in production of defective hormone, receptor or post-receptor signalling (Kiyama and Wada-Kiyama 2015; Söder 2016). The disruption of endocrine functions will lead to a multitude of disorders, which may manifest immediately or have delayed onset (Söder 2016; Maqbool et al. 2016).

Endocrine disrupting compounds can play agonistic roles by stimulating hormone production but not at the right time (Darbre 2015). If the stimulation is at the right time, such stimulation will be excessive. Endocrine disruptors can mimic normal hormones by binding to hormonal receptors and initiate normal responses at the wrong time (Combarnous and Nguyen 2019). In antagonistic action, EDCs may modify the binding site of a normal hormone by binding to hormonal receptors but not activate it (Nguyen 2018). In this type, the normal hormone is prevented from binding to its site since an EDC has occupied it (Rosenfeld and Cooke 2019). EDCs may bind to hormone carrier or transport proteins in the blood thereby reducing the hormones in circulation (Rosenfeld and Cooke 2019). Another way by which EDCs affect the body is to interfere with the metabolic processes by affecting the rate of synthesis or breakdown of natural hormones and disrupt the actions of enzymes involved in steroidogenesis (Yang et al. 2015). Bisphenol A (BPA) and nonylphenol (NP) can compete with E2 in binding to oestrogen receptors with a similar preference and degree at nanogram concentration (Kuiper et al. 1998). BPA and NP can disrupt androgen hormonal functions and act as potent anti-androgen receptor (AR) antagonists. They can affect multiple steps in the activation and functions of androgen receptors, thereby inhibiting the binding of native androgens to their receptors, hinder interaction with its coregulator and its subsequent transactivation (Wang et al. 2017; Kuiper et al. 1998).

In the aquatic environments, the presence of some EDCs (alkylphenols, phytoestrogens and oestrogens) in conjunction with hydrodynamic factors such as temperature has been reported to promote eutrophication in freshwater (Rocha et al. 2014). Jia et al. (2019) studied cyanobloom in freshwater and detected 29 EDCs promoting eutrophication. 17β-estradiol (E2) is a natural hormone in women that promotes secondary sexual characters. Its synthetic form, 17α-ethynylestradiol (EE2), is present in birth control pills and it is the most widely used contraceptive (Evans and Sutton 2015). When EE2 is released into the environment, it can induce feminization, hypogonadism and sexual dysfunction in male organisms (Kuhl 2005). 2,4-Dichlorophenol (2,4-D) is a component of herbicides, antimicrobials, nematicides and some pharmaceuticals (Park and Kim 2018) from where it gets into the environment. It exhibits anti-androgen activities and reduces the oestrogen levels in the female (Li et al. 2012a, b). Triclosan is a broad-spectrum antimicrobial, present as one of the main components in many pharmaceuticals, personal care products, household products such as beddings, dish-washing products and sporting items from where it gets into the environment through wastewater (Dhillon et al. 2015). Triclosan and its metabolites had been isolated in human fluids including breast milk (Bever et al. 2018). Triclosan has been to shown to induce overall depression of the central nervous system in mice, decrease sperm count in male rats, and malformations in foetal development (James et al. 2010; Dhillon et al. 2015). Long exposure to triclosan in mice has been shown to enhance hepatocellular carcinoma (Yeah et al. 2014). Many of the pesticides used in farming activities, ranging from herbicides to antimicrobial chemicals are implicated in endocrine disruption (Gaudriault et al. 2017; Wong et al. 2019). Atrazine was the most widely used herbicide against broadleaf weeds before it was banned in 2003 in the USA and Europe because it was ubiquitous in drinking water (Székács et al. 2015). The presence of atrazine in drinking water has been linked to feminization of male gonads (Hayes et al. 2011; U.S. Environmental Protection Agency 2007). It may remain in the soil up to 4 years and might be washed to rivers or leached to groundwater where it degrades slowly (U.S. Environmental Protection Agency 2007). There are various reports of the effects of EDCs on wildlife, especially in freshwater ecosystems, such as abnormal development and death of embryos (Arukwe et al. 2016; Ortiz-Villanueva et al. 2018), changes in sexual behaviour (Kanda 2019), feminization of male animals (Carnevali et al. 2018) and altered immune functions (Nowak et al. 2019). The effects of EDCs had also been recorded in birds, especially those feeding in polluted waters (Roman et al. 2019; Jessl et al. 2018) and may ultimately lead to loss of biodiversity.

The sources of EDCs in the environment include municipal and household wastewater, building materials, agricultural run-off, mining, industrial emissions and solid wastes. They may not be effectively removed at the wastewater treatment plants (WWTPs) from where they find their ways to the receiving water bodies where other organisms pick them up (Rogowska et al. 2019; Vega-Morales et al. 2013; Zhou et al. 2019). EDCs such as phthalates and triclosan in personal care products such as cosmetics, lotions, fragrances and soaps contain, get into the environment through wastewater (Magueresse-Battistoni et al. 2017; Nicolopoulou-Stamati et al. 2015). Food and water are the major routes of exposure to EDCs (Wee and Aris 2019; Scialabba 2019; Russo et al. 2019). A variety of EDCs have been observed in the treated drinking water supply throughout the world, particularly in tap water from as low as 0.2 ng/L to as high as 5510.0 ng/L, while a maximum concentration (28,000.0 ng/L) was observed in drinking water from the wells in India (Wee and Aris 2019). Some food packaging materials such as plastics contain EDCs (Benjamin et al. 2017; Hejmej et al. 2011). Children are more vulnerable because some of the toys and feeding bottles contain EDCs (Wong and Durrani 2017). Some processed foods carry some EDCs from manufacturing processes and some preservatives added to such foods have endocrine disruptive abilities (Maffini et al. 2016). Agrochemicals such as pesticides and livestock drugs and hormones are implicated in contributing to the environmental EDCs load with products like atrazine and 2,4-Dichlorophenol being the highest (Székács et al. 2015).

It has been known that the effects of EDCs are not restricted to the localities where they are generated because they can travel rapidly through the food chain; spread by running water and transported by the wind far beyond the point of release (WHO 2012). The persistence of some EDCs in the environment is related to their structural stability, which made it easy for them to pass from one level of the food chain to another and bioaccumulate (Kudłak et al. 2015). They can accumulate in the fatty and other tissues in the body of animals (Lv et al. 2019; Zhou et al. 2019). This study investigated the concentrations of estrone, 17β-estradiol, bisphenol A, triclosan, imidazole, triazole, 2,4-dichlorophenol, nonylphenol, atrazine and 4-octylphenol in four rivers of economic importance in the Eastern Cape Province of South Africa. The aim was to determine the concentrations and sources of the selected compounds in the rivers so as to aid in the proper management of the aquatic environment and wastewaters effluents. Wastewater influents and the effluents released to these rivers were analysed to determine the sources of these compounds in the rivers.

## Materials and methods

### Study area

**Fig. 1:**
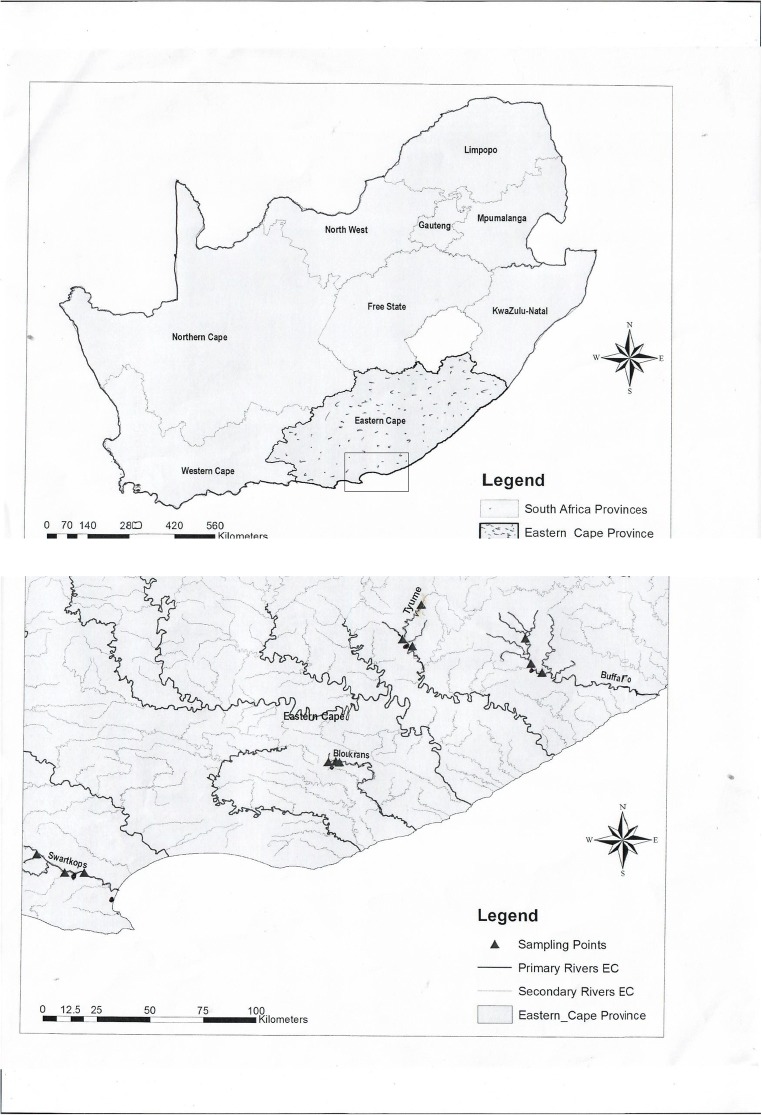
Map of South Africa showing the sampling sites

The four major rivers sampled in Eastern Cape Province of South Africa in this study include Bloukrans (upstream, 33^0^ 19′ 0.07″S; 26^0^ 31′ 20.9″E; midstream: 33^0^ 18′51.4″S, 26^0^ 33′11.5″ E and downstream: 33^0^ 19′ 07.1″ S, 26 ^0^ 34′ 05.7″ E), Tyhume (upstream, 32^0^ 36′ 38.72″ S; 26^0^ 54′ 34.15″ E; midstream, 32^0^ 47′ 42.95″ S, 26^0^ 50′ 88″ E and downstream, 32^0^ 50′ 15″ S, 26^0^ 53′ 31.27″ E.), Buffalo (upstream, 32^0^4 7′ 23.74″S, 27^0^ 22′ 10.56″ E; midstream, 32^0^ 53′ 49.14″ S, 27^0^ 23′ 34.08″ E and downstream: 32^0^56′3.6” S; 27^0^ 26′ 25.18″ E) and Swartikops (upstream, 33^0^ 42′ 59.64″ South (S), 25^0^ 17′ 16.43″ East (E); midstream 33^0^ 47′ 31.08″ S, 25^0^ 24′ 26.96″ E and downstream, 33^0^ 47′ 31.92″ S; 25^0^ 29′26.26″ E). Figure 1 shows the map of South Africa with the sampling sites. Upstream samples were collected close to the river sources, midstream after the rivers had passed through major towns and downstream after receiving wastewater effluents. The four wastewater treatment plants (WWTP) discharging treated effluents to these rivers include Belmont Wastewater Treatment Works, Grahamstown, discharging to Bloukrans River. Grahamstown has over 80,000 inhabitants (Department of Statistics 2019). Fort Hare WWTP in Alice, discharging to Tyhume River. Alice has over 127,000 inhabitants as at mid-year 2019 (Department of Statistics 2019). Zwelitsha Wastewater Treatment Works in King William’s Town, discharging to Buffalo River. King Williams Town has over 227,000 inhabitants (Department of Statistics 2019). Kelvin Jones WWTP in Uitenhage discharging in to Swartkops River. Uitenhage has over 71,000 inhabitants (Department of Statistics 2019). This study was carried out in the year 2018. Triplicate samples of water were collected from each sample site established at upstream, midstream and downstream reaches of the rivers. Wastewater influents and effluents samples were collected from their respective WWTPs.

### Materials

All chemicals used were of HPLC grade and purchased from Sigma-Aldrich (Johannesburg, South Africa). These include acetone, methanol, nonylphenol (Technical grade), dichlorophenol (99%), estrone (99%), 17β- estradiol (98%), bisphenol A (97%), octylphenol (99%), triclosan (99%), atrazine (99%), (99%) and 1,2,4-and triazole (98%). De-ionized water was produced with Millipore (Millipore SA, France). Freezing was on a rotary evaporator (Büchi Rotavapor R-210 with Büchi Bath B-491, Büchi Labotechnik, Switzerland). Drying of the frozen water samples was with Vir Tis BenchTop K freeze dryer, equipped with Elnor vacuum pump (SP Scientific, Pennsylvania USA). Solid-phase extraction (SPE) tubes (Supelclean LC-18) and vacuum manifold (Visiprep) were purchased from Sigma-Aldrich (Johannesburg, South Africa).

### Procedure

Sample bottles (1 l) were prepared by washing in phosphate-free soap, rinsing with deionised water, dry and soaked into acetone for 30 min, rinsed with hexane and dried at 120 °C (Olujimi et al. 2012). The bottles were rinsed three times with sample water at the point of collection. One litre water sample was collected in triplicate into the prepared bottle at each sample site. The bottles were filled with water samples, tightly covered and preserved in ice-box to limit the activities of microorganisms during transportation. The samples were transported to the laboratory for analyses. Extraction of the organic content of the water samples was done within 24 h of collection. 300 ml of each sample was frozen in liquid nitrogen placed in the water bath mounted on the rotary evaporator. Frozen water samples were transferred to a drier fitted with a vacuum pump to evaporate the water content. The samples were dissolved in acetone and filtered to extract the organic compounds. The extraction was repeated with methanol. The filtrates from both solvents were combined to a labelled vial. The vials were allowed to dry in an oven set at 30 °C. The dried filtrates were re-dissolved in deionised water before transferred to labelled SPE tubes for solid-phase extraction. Deionised water was used for the control experiment instead of sample water.

Extraction of compounds of interest was with disposable LC-18 solid-phase extraction (SPE) columns (Olujimi et al. 2010; Neale et al. 2018). The SPE columns were conditioned with 5 ml methanol and rinsed with deionised water (Minh et al. 2016). The re-dissolved water extracts were passed through the SPE tube mounted on Supelco vacuum manifold connected to a vacuum pump. Sample flow was regulated to 15 drops per minute. The tubes were rinsed with deionised water before elution. Methanol was used to elute the compounds from the SPE tubes (Lv et al. 2016). Elutes were collected into glass vials and carefully labelled for LC-MS analysis.

### Sample analysis

Liquid chromatography coupled with mass spectrometry (LC-MS) analysis was according to the method described by Petrie et al. (2016) and Archer et al. (2017). Lyophilized samples were reconstituted in 9 ml of 10% MeOH, together with 1 ml of 50 μg/L p-aminosalicylic acid (PAS) as the internal standard. The entire 10 ml sample was then passed through HLB SPE cartridge (Waters, Milford, USA), washed with water and the analytes eluted using 1 ml methanol. Chromatography was performed on Waters Acquity ultra-high-performance liquid chromatography (UPLC), using 0.1% formic acid in water, and 0.1% formic acid in acetonitrile as mobile phases. The UPLC column was ethylene bridge hybrid (BEH) C18, 2.1x100mm and 1.7 μm. The UPLC was coupled to Xevo tandem quadrupole spectrometer (TQ-S) forming UPLC-MS/MS, for quantitative and qualitative analyses of samples. The instrument was operated in the positive electrospray ionization (ESI) mode with the reaction transitions monitored for each component for quantification and identification, respectively. The instrument pressure was maintained within 0–12,000 psi. The method was validated in-house; matrix-matched calibration graphs were prepared and good linearity (r^2^ > 0.99) was achieved over the concentration ranges tested for each compound. Analyte recoveries during HLB sample cleanup were 103% ± 6.9. Table 1 shows the characteristics of the EDCs with the limit of detection (LOD) of the samples in LC-MS/MS.

**Table 1:**
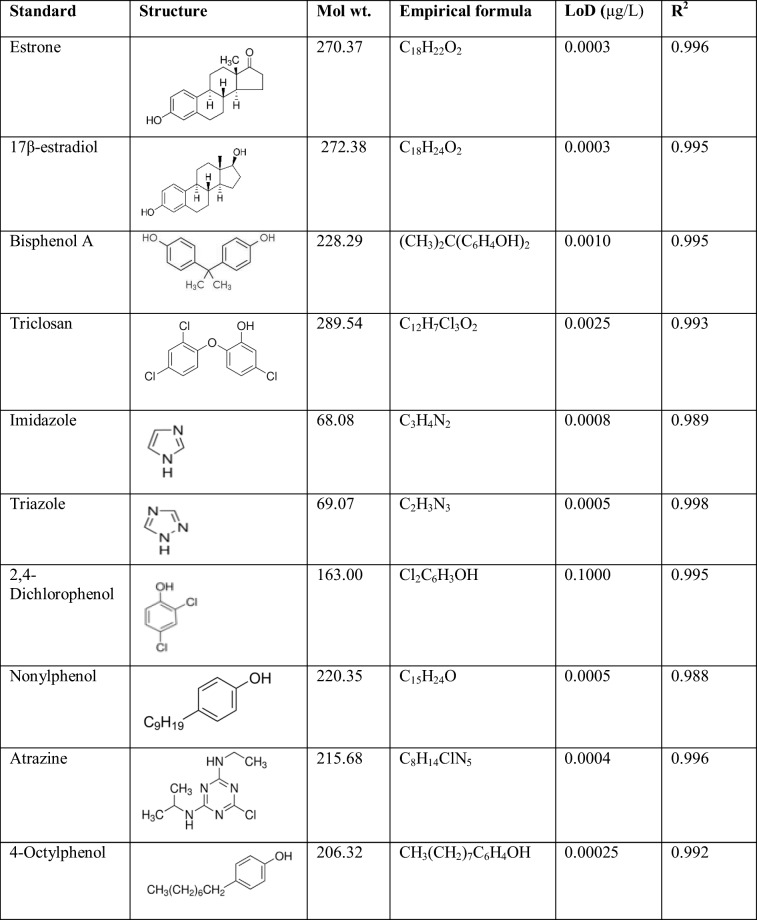
Properties and analytical limits for the target compounds

## Results

**Fig. 2:**
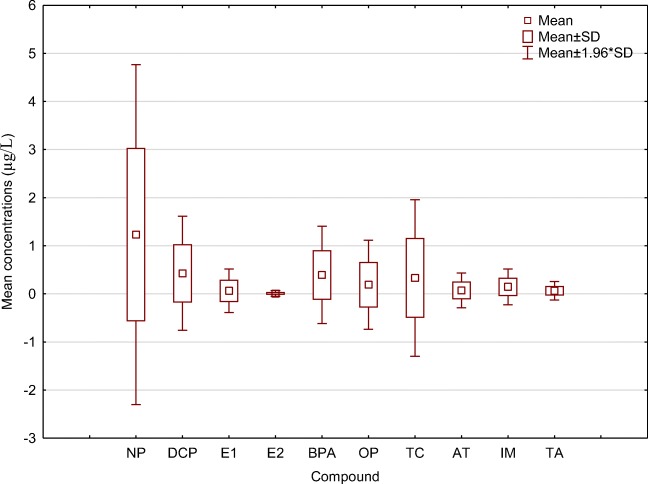
Box plot for the concentrations of the compounds in the samples. *NP* nonylphenol, *DCP* 2,4-dichlorophenol, *E1* estrone, *E2* 17β-estradiol, *BPA* bisphenol A, *OP* 4-octylphenol, *TC* triclosan, *AT* atrazine, *IM* imidazole, *TA* triazole

The overall mean concentrations of the compounds in the waters sampled were presented in Fig. 2. Results show that nonylphenol (NP) has the highest concentration in the samples with a mean of 1.297 μg/L, followed by dichlorophenol (DCP) with a mean concentration of 0.449 μg/L and bisphenol A (BPA) with 0.415 μg/L. The least being 17β-oestradiol (E2) with 0.0095 μg/L. Generally, all the wastewater samples showed higher concentrations of nonylphenol (NP) than other samples (Table 2). The concentrations were reduced in their corresponding treated effluents except for GE that retained 58% of NP in its effluents. King Williams Town wastewater influents (KW) had the highest mean concentration of NP with 6.72 μg/L, but only 5.7% was retained in the effluents (KE). Alice wastewater influents (AW) had 3.131 μg/L but 11.4% was retained in the treated effluents (AE). Bloukrans midstream samples (BM) had the highest mean concentration of NP (2.553 μg/L) amongst the freshwater samples. This might have influenced its downstream (BD) samples with a mean concentration of 2.456 μg/L.

**Table 2:** Concentrations of the compounds in the samples (mean values are μg/L with standard deviation, Sd). Values with < LoD are below the limit of detection. NA = not applicable

Sample		NP	DCP	E1	E2	BPA	OP	TC	AT	IM	TA
BU	Mean	0.0627	0.0107	0.0017	0.0163	0.0173	< LoD	< LoD	0.0207	< LoD	< LoD
	Sd	0.0023	0.0023	0.0002	0.0023	0.0023	NA	NA	0.0023	NA	NA
BM	Mean	2.5533	0.7373	0.0613	< LoD	0.4650	< LoD	0.1469	< LoD	0.5737	0.1810
	Sd	0.0841	0.0841	0.0042	NA	0.0841	NA	0.0134	NA	0.0841	0.0174
BD	Mean	2.4560	0.4920	0.0403	0.0075	0.4770	0.0850	0.0623	< LoD	< LoD	0.0505
	Sd	0.1424	0.1424	0.0078	0.0012	0.0358	0.0091	0.0091	NA	NA	0.0076
GW	Mean	4.3773	0.8540	0.0263	0.0113	1.4683	0.0357	0.2323	0.8123	0.6187	0.1490
	Sd	0.2874	0.2874	0.0027	0.0021	0.0874	0.0027	0.0274	0.0274	0.0874	0.0207
GE	Mean	2.5557	< LoD	0.0151	< LoD	0.3451	< LoD	0.0446	< LoD	0.0950	0.0943
	Sd	0.0808	NA	0.0019	NA	0.0346	NA	0.0084	NA	0.0141	0.0086
FU	Mean	0.1017	0.0353	0.0054	< LoD	0.0465	< LoD	< LoD	< LoD	< LoD	0.0392
	Sd	0.0095	0.0031	0.0003	NA	0.0076	NA	NA	NA	NA	0.0055
FM	Mean	0.1962	0.0312	0.0000	< LoD	0.0943	< LoD	< LoD	< LoD	0.0213	0.0061
	Sd	0.0208	0.0026	NA	NA	0.0118	NA	NA	NA	0.0028	0.0014
FD	Mean	0.1454	0.2971	0.0013	< LoD	0.0185	< LoD	< LoD	< LoD	0.1788	0.0130
	Sd	0.0200	0.0200	0.0001	NA	0.0028	NA	NA	NA	0.0170	0.0013
FM	Mean	0.1962	0.0312	< LoD	< LoD	0.0943	< LoD	< LoD	< LoD	0.0213	0.0061
	Sd	0.0208	0.0026	NA	NA	0.0118	NA	NA	NA	0.0028	0.0014
FD	Mean	0.1454	0.2971	0.0013	< LoD	0.0185	< LoD	< LoD	< LoD	0.1788	0.0130
	Sd	0.0200	0.0200	0.0001	NA	0.0028	NA	NA	NA	0.0170	0.0013
KW	Mean	6.7197	1.7190	0.0124	0.0061	1.0110	0.0572	0.1767	0.1510	0.2665	0.4290
	Sd	0.1669	0.0367	0.0022	0.0007	0.0367	0.0058	0.0093	0.0093	0.0137	0.0147
KE	Mean	0.3840	0.7113	< LoD	< LoD	0.0181	< LoD	0.0410	0.0123	0.0174	0.0273
	Sd	0.0253	0.0340	NA	NA	0.0021	NA	0.0027	0.0003	0.0035	0.0051
SU	Mean	0.0312	0.0067	< LoD	< LoD	0.0067	< LoD	< LoD	< LoD	0.0119	< LoD
	Sd	0.0032	0.0010	NA	NA	0.0008	NA	NA	NA	0.0021	NA
SM	Mean	0.1640	0.0963	0.0020	< LoD	0.3417	1.4533	< LoD	0.0446	0.2480	0.0187
	Sd	0.0107	0.0107	NA	NA	0.0227	0.0253	NA	0.0044	0.0207	0.0041
SD	Mean	0.3337	0.1273	0.0020	< LoD	0.3117	0.4013	2.7147	0.0203	< LoD	0.0260
	Sd	0.0214	0.0073	NA	NA	0.0148	0.0140	0.0373	0.0039	NA	0.0033
UE	Mean	0.1430	0.8090	0.0134	0.0025	1.6837	0.0470	0.0827	0.0095	0.2055	0.0673
	Sd	0.0087	0.0200	0.0010	0.0004	0.0553	0.0080	0.0045	0.0020	0.0110	0.0062
TU	Mean	0.1293	0.0130	< LoD	< LoD	0.0327	< LoD	< LoD	< LoD	0.0150	0.0170
	Sd	0.0073	0.0013	NA	NA	0.0047	NA	NA	NA	0.0007	0.0013
TM	Mean	0.2141	0.0263	0.0009	< LoD	0.1173	< LoD	< LoD	< LoD	0.0897	0.0207
	Sd	0.0100	0.0034	0.0001	NA	0.0113	NA	NA	NA	0.0085	0.0047
TD	Mean	0.5867	0.2613	< LoD	< LoD	0.0177	< LoD	0.0097	< LoD	< LoD	< LoD
	Sd	0.0187	0.0280	NA	NA	0.0035	NA	0.0018	NA	NA	NA
AW	Mean	3.1307	2.2000	1.0600	0.1350	1.1927	0.0193	2.8563	0.2560	0.3717	0.1013
	Sd	0.0807	0.0487	0.0267	0.0093	0.0447	0.0012	0.1900	0.0153	0.0207	0.0095
AE	Mean	0.3580	0.1180	0.0131	0.0026	0.2187	1.6827	0.2743	0.1184	0.2027	0.0153
	Sd	0.0400	0.0093	0.0043	0.0004	0.0141	0.0300	0.0180	0.0125	0.0072	0.0025
CTR	Mean	< LoD	< LoD	< LoD	< LoD	< LoD	< LoD	< LoD	< LoD	< LoD	< LoD

*NP* nonylphenol, *DCP* dichlorophenol, *E1* estrone, *E2* 17β oestradiol, *BPA* bisphenol A, *OP* octylphenol, *TC* triclosan, *AT* atrazine, *IM* imidazole, *TA* triazole

*BU* bloukrans upstream, *BM* bloukrans midstream, *BD* bloukrans downstream; *FU* buffalo upstream, *FM* buffalo midstream, *FD* buffalo downstream; *SU* swartkops upstream, *SM* swartkops midstream, *SD* swartkops downstream; *TU* tyhume upstream, *TM* tyhume midstream, *TD* tyhume downstream; *GW* grahamstown wastewater, GE grahamstown treated effluents; *KW* king Williams wastewater, *KE* king Williams treated effluents; *AW* alice wastewater, *AE* alice treated effluents; *UE* uitenhage treated effluents, *CTR* control

Dichlorophenol (DCP) has its highest mean concentration of 2.200 μg/L in AW but only 5.36% of it was present in the treated effluents AE (Table 2). Sample KW has 1.719 μg/L of DCP but 41.38% escaped the treatment plant into the treated effluents. DCP was below the detection limit in Grahamstown effluent (GE) samples. Bloukrans River midstream (BM) samples had a mean concentration of 0.737 μg/L DCP while its downstream (BD) samples had 0.492 μg/L. Swartkops River samples SD and TD had mean concentrations of 0.127 and 0.261 μg/L, respectively. Olujimi et al. (2012) recorded various concentrations of DCP in different South African freshwater, wastewater and treated effluents. Zhong et al. (2018) recorded an average of 1.56 μg/L of DCP in a Chinese river.

The concentrations of bisphenol A (BPA) is shown in Table 2. BPA is present in all the samples at different concentrations. Its concentrations were lower in the upstream samples (BU, FU, SU and TU) than other reaches of the rivers. Uitenhage treated effluents (UE) had the highest mean concentration of 1.684 μ/L, followed by GW with 1.468 μg/L. Uitenhage wastewater cannot be accessed due to restricted permission. Grahamstown treated effluents (GE) contained 23.50% of BPA unremoved, while Alice effluents (AE) had 18.34%. Wanda et al. (2017) reported various concentrations of BPA in some South African rivers. BPA has been detected in rivers and wastewaters in many countries of the world such as India, China, Russia and several others and similarly its concentrations in wastewater effluents were reduced compared to influents (Corrales et al. 2015).

The mammalian hormones, estrone (E1) and 17β-estradiol (E2) were detected in most of the samples with E1 more common than E2 (Table 2). Both were less concentrated in the samples compared to other compounds. Alice wastewater influents (AW) had the highest mean concentrations of both hormones, with 1.06 μg/L of E1 and 0.135 μg/L of E2 (Table 2). Bloukrans River midstream (BM) samples had the second-highest concentration of E1 with 0.062 μg/L. It was observed that 57% of E1 was not removed from GW during treatment and hence present in the effluents (GE). The WWTP was able to remove > 98% E1 from AW. These hormones were reported in the environment in China (Huang et al. 2014), Australia (Leusch et al. 2006) and the European Danube River (König et al. 2017) amongst others.

Alice wastewater influents (AW) had the highest mean concentration of octylphenol (OP) with 1.683 μg/L. Only 1% of it was present in its effluents. Other wastewaters had their OP totally removed during treatment. The concentrations of OP in the midstream (SM) and downstream (SD) samples of Swartkops River were 1.453 μg/L and 0.4 μg/L, respectively. OP was below the limit of detection in all the upstream samples of the rivers (Table 2). Alice wastewater influents (AW) samples had the highest mean concentration of triclosan with 2.856 μg/L, while Swartkops downstream sample SD with 2.715 μg/L. The retention of OP in wastewater effluents (19% in sample GE, 23% in KE and 9.6% in AE) was an indication that it was not totally removed during wastewater treatment. It was below the limit of detection in all the upstream samples (BU, FU, SU and TU), but present in the midstream and downstream samples of Bloukrans and Swartkops Rivers, and downstream sample of Tyhume River. This compound has been reported in wastewaters and treated effluents around the world (Thomaidi et al. 2017; Madikizela et al. 2014; Wang et al. 2014).

The mean concentration of atrazine (AT) in Grahamstown wastewater influents (GW) was 0.812 μg/L, while it was below the limit of detection in its effluents (GE). Alice wastewater influents (AW) had a mean concentration of 0.256 μg/L, with 0.122 μg/L or 47.5% retained in its effluents (AE). Sample KW has 0.141 μg/L of AT with 7.95% retained in its effluents (KE). Atrazine is below the limit of detection in upstream samples except BU with 20.7 μg/L.

Imidazole (IM) is present in all the wastewater samples and the treated effluents. Its concentration was highest in Grahamstown wastewaters influents (GW) with a mean value of 0.619 μg/L and its effluents had 15.24% of the compound retained. Alice wastewater influents (AW) had 0.372 μg/L of IM and its treated effluents (AE) had 0.203 μg/L or 54.53% retained in the effluents. Sample KW has IM concentration of 0.267 μg/L with 6.64% retained in its treated effluents (KE). Midstream samples BM and SM have IM concentrations of 0.574 and 0.248 μg/L, respectively, an indication of sources other than WWTPs. Triazole (TA) was detected in all the samples except BU, SU and TD. It has its highest mean concentration in King Williams Town wastewater influents (KW) with 0.429 μg/L and 6.29% of it escaped WWTP into the effluents. Grahamstown wastewater influents (GW) had a mean concentration 0.149 μg/L of TA with 0.094 μg/L or 63.29% present in their treated effluents. Alice wastewater influents contain 0.101 μg/L of TA with 15.15% retained in the effluents (AE). Amongst the freshwater samples, Bloukrans midstream samples ranked highest with mean TA concentration of 0.181 μg/L, followed by its downstream sample (BD) with 0.051 μg/L.

## Discussion

The concentration of nonylphenol may be explained with its uses in manufacturing of household products such as detergents, emulsifiers, antioxidants, paint, pesticides, personal care products, plastics, solubilisers and as lubricating oil additives (Kovarova et al. 2013). It is rated as third largest surfactant worldwide (Brunelli 2018). It can also be formed from the anaerobic breakdown of ethoxylated alkylphenols (Araujo et al. 2018). NP affects reproduction, fertility and infant size at birth (Huang et al. 2017). NP has been reported severally in WWTPS and surface waters worldwide (Chokwe et al. 2016; Carvalho et al. 2016; Belhaj et al. 2015). It has also been reported that WWTPs were not able to remove it totally from the wastewater thereby polluting the receiving rivers through treated effluents (Fleming et al. 2016; Belhaj et al. 2015; Dotan et al. 2016). Dichlorophenol is used in the production of antimicrobials, herbicides and some pharmaceuticals (Park and Kim 2018). It is known to affect androgen and oestrogen secretion in both male and female animals (Li et al. 2012a, b). The presence of DCP in the midstream samples shows that this compound enters the rivers from sources other than the WWTPs. These sources may be city and agricultural runoffs and waste dump. Bisphenol is a constituent of plastics and plastic products with the ability to mimic oestrogen and prevent the normal binding of the hormone thereby causing infertility, obesity and cancer (Saal et al. 2012; Mirmira and Evans-Molina 2014). The presence of BPA in the midstream samples (BM, FM, SM and TM) of the rivers might result from a combination of waste dump, especially improperly disposed plastic packagings and runoff. Sub-lethal doses of bisphenol A in the aquatic environment have been shown to have adverse effects on the developing zebrafish embryos (Ortiz-Villanueva et al. 2018). BPA has been implicated in infertility, obesity, cancer and neurologic impairments (Saal et al. 2012; Itoh et al. 2012; Mirmira and Evans-Molina 2014). These observations earned BPA a place amongst chemicals of high concern and subsequently banned in baby products (Mirmira and Evans-Molina 2014).

The presence of oestrone and 17β oestradiol in Alice wastewater influents may be due to the input from the nearby university community. E1 is present in some drugs as menopausal hormonal supplement and virginal creams for women (Friel et al. 2005; Searchlight Pharma Inc 2016) and it is also excreted with urine as estrone sulphate (Kuhl 2005), which might have contributed to its presence in the environment. The observed concentration of the hormones in Bloukrans River samples might be suggesting that either most of the household wastewater of Grahamstown were not passing through the sewers or the free-range cattle, grazing around the Belmont valley (where the rivers passes) contributed to the hormones in the samples or both. Fatoki et al. (2018) corroborated the fact that livestock is a source of oestrogen in the environment. Octylphenol is used in the manufacture of detergents, emulsifiers, spermicides and contraceptives (Ripamonti et al. 2018). This might be the reason for its presence in the wastewaters influents.

Triclosan is used in the manufacturing of some pharmaceuticals, personal care and household products from where it gets into the wastewater (Dhillon et al. 2015). Atrazine is one of the top 25 chemicals used as pesticide in South Africa, being the active ingredient in glyphosate and other herbicides; it has high mobility the environment, this might account for its presence in the samples (Dabrowski et al. 2014). BU is from upstream of the Bloukrans River where a recreational resort is located. The presence of atrazine in this sample might be as result of the chemicals used in maintaining the lawns. Imidazole is commonly used in manufacturing of drugs, dye, photographic chemicals, polyurethanes and corrosion inhibitors (Spasiano et al. 2016). Imidazole based drugs had been reported to be present in wastewater and receiving rivers in several countries (Mirzaei et al. 2019; Wang et al. 2018). Trizoles are components of drugs, light stabilizers, chemosensors and corrosion retarding agents (Rani 2014; Ceesay et al. 2016). They have been reported as pollutants in wastewaters and receiving rivers (Huang et al. 2013; Vimalkumar et al. 2018).

## Conclusion

All the ten EDCs investigated in this study were present in most of the water samples with nonylphenol having the highest mean concentration. Octylphenol, triclosan and atrazine concentrations were below detection limits in some samples. The upstream samples of the rivers showed the least concentrations of the compounds and in most cases were below detection limits. Concentrations of these compounds in the midstream samples suggested other sources of environmental EDCs such as open dump of wastes and runoffs from adjacent farmlands. It was observed that WWTPs did not achieve total removal of EDCs from the wastewater influents, thereby serving as sources of EDCs pollution in the receiving rivers. Better technology input into wastewater treatment is necessary to achieve total removal of these compounds. Environmental education is needed for the communities on proper waste disposal.
